# Supplementary material for: Repeated upslope biome shifts in Saxifraga during late-Cenozoic climate cooling
Source: Nat Commun. 2024 Feb 6;15:1100. doi: 10.1038/s41467-024-45289-w (PMC10847498; doi:10.1038/s41467-024-45289-w)
Supplement: Supplementary file 1 — Supplementary Information [file 41467_2024_45289_MOESM1_ESM.pdf]

TITLE

Repeated upslope biome shifts in *Saxifraga* during late-Cenozoic climate cooling

AUTHOR LIST

Tom Carruthers[1,+], Michelangelo S. Moerland[1,2,+], Jana Ebersbach[3,4], Adrien Favre[5], Ryan A. Folk[6], Julie A. Hawkins[2], Alexandra N. Muellner-Riehl[3,4], Martin Röser[7], Douglas E. Soltis[8,9], Natalia Tkach[7], William J. Baker[1,†], Jurriaan M. de Vos[10,†], Wolf L. Eiserhardt[1,11,†]

[+] These authors contributed equally

[†] These authors jointly supervised the work

Corresponding author: Wolf L. Eiserhardt, wolf.eiserhardt@bio.au.dk

AFFILIATIONS

[1] Royal Botanic Gardens, Kew, Richmond, Surrey, TW9 3AE, United Kingdom.

[2] School of Biological Sciences, University of Reading, Whiteknights, Reading, Berkshire. RG6 6EX. United Kingdom.

[3] Department of Molecular Evolution and Plant Systematics & Herbarium (LZ), Institute of Biology, Leipzig University, D-04103 Leipzig, Germany.

[4] German Centre for Integrative Biodiversity Research (iDiv) Halle-Jena-Leipzip, D-04103, Leipzig, Germany.

[5] Regional Nature Park of the Trient Valley, la Place 24, 1922 Salvan, Switzerland.

[6] Department of Biological Sciences, Mississippi State University, Mississippi State, MS, USA 39762.

[7] Martin Luther University Halle-Wittenberg, Institute of Biology, Geobotany and Botanical Garden, Dept. of Systematic Botany, Neuwerk 21, 06108 Halle, Germany.

[8] Florida Museum of Natural History, University of Florida, Gainesville, FL, USA 32611.

[9] Department of Biology, University of Florida, Gainesville, FL, USA 32611.

[10] Department of Environmental Sciences - Botany, University of Basel, Schönbeinstrasse 6, 4056 Basel, Switzerland.

[11] Department of Biology, Aarhus University, 8000 Aarhus C, Denmark.

Supplementary Figure 1

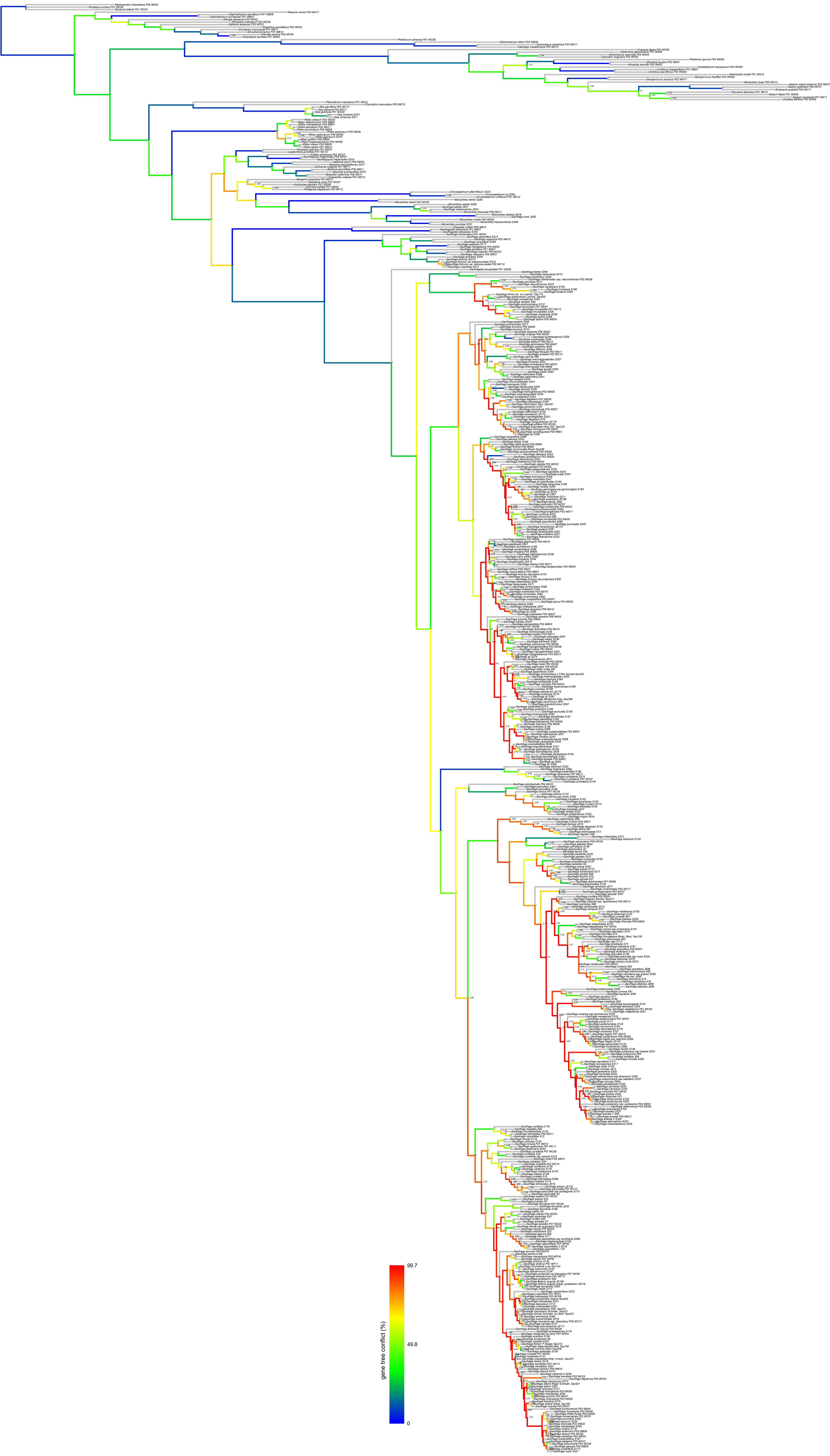

Supplementary Figure 1. Estimated species tree topology from ASTRAL. Node labels refer to the local posterior support. Branch colours correspond to the level of gene tree conflict (% of gene trees that do not share a bipartition that is congruent with the species tree branch).

Supplementary Figure 2a

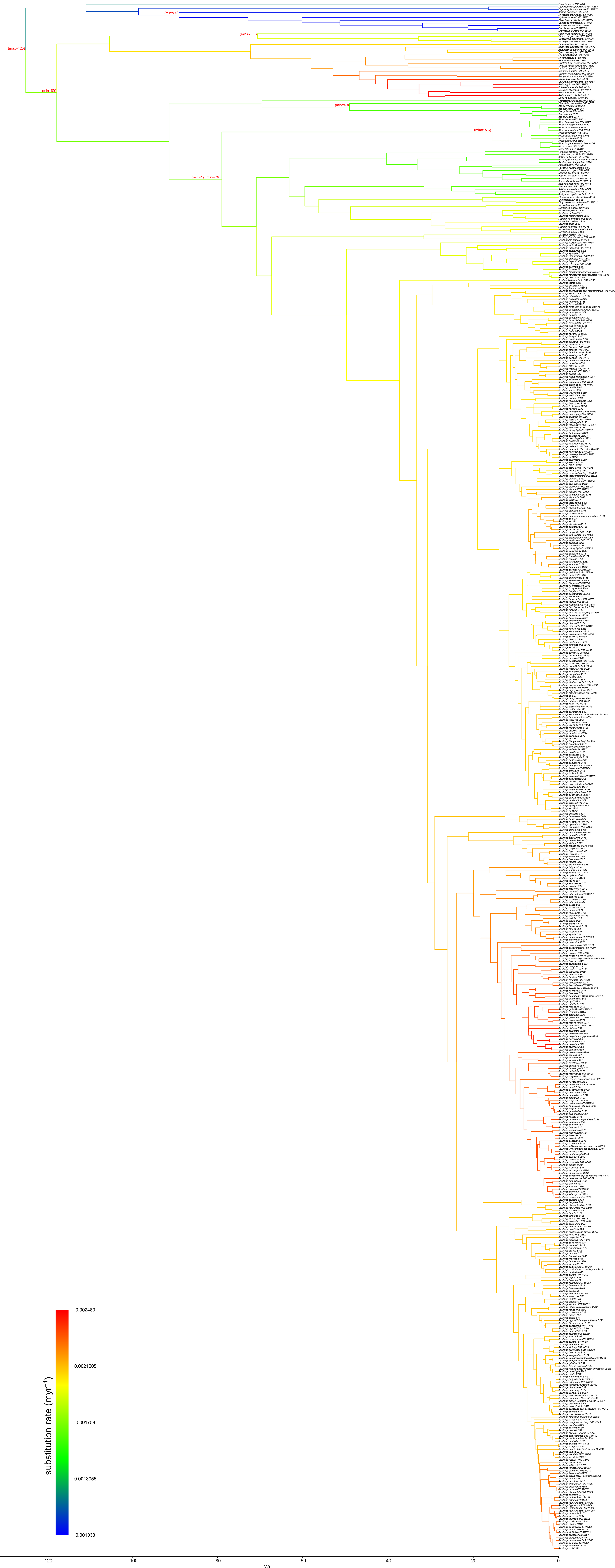

Supplementary Figure 2b

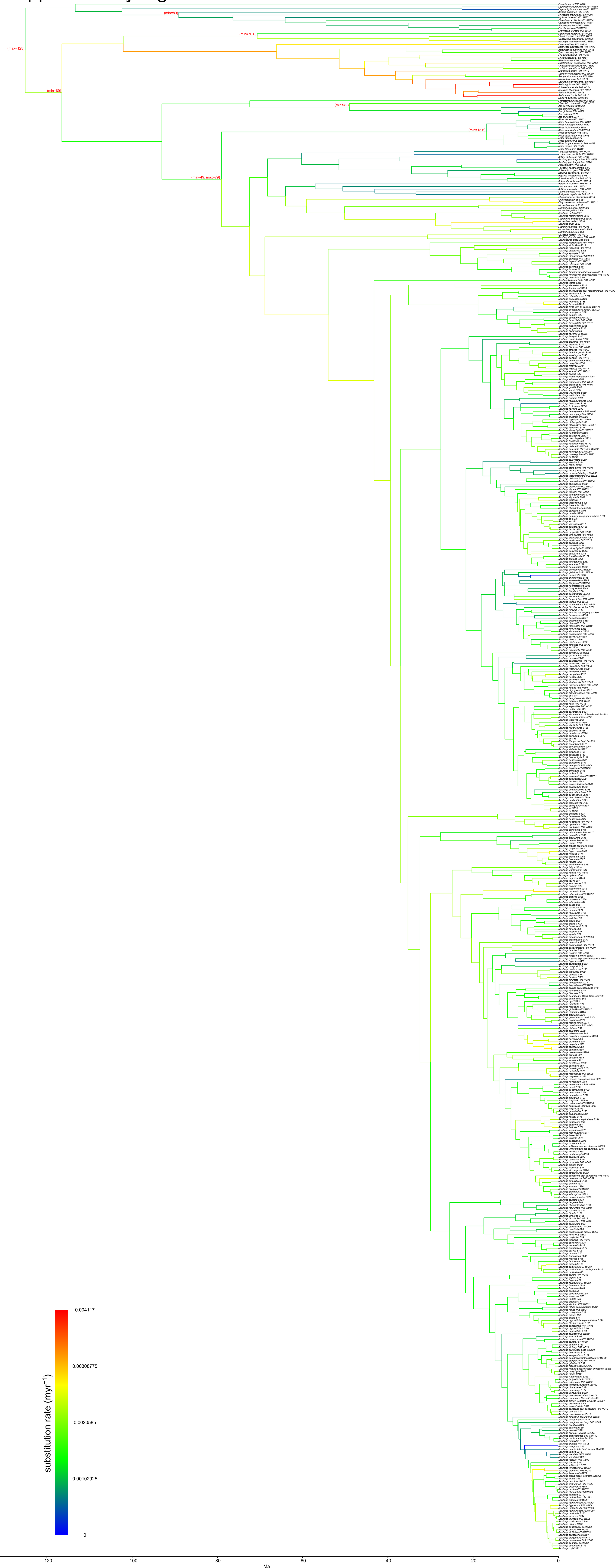

Supplementary Figure 2c

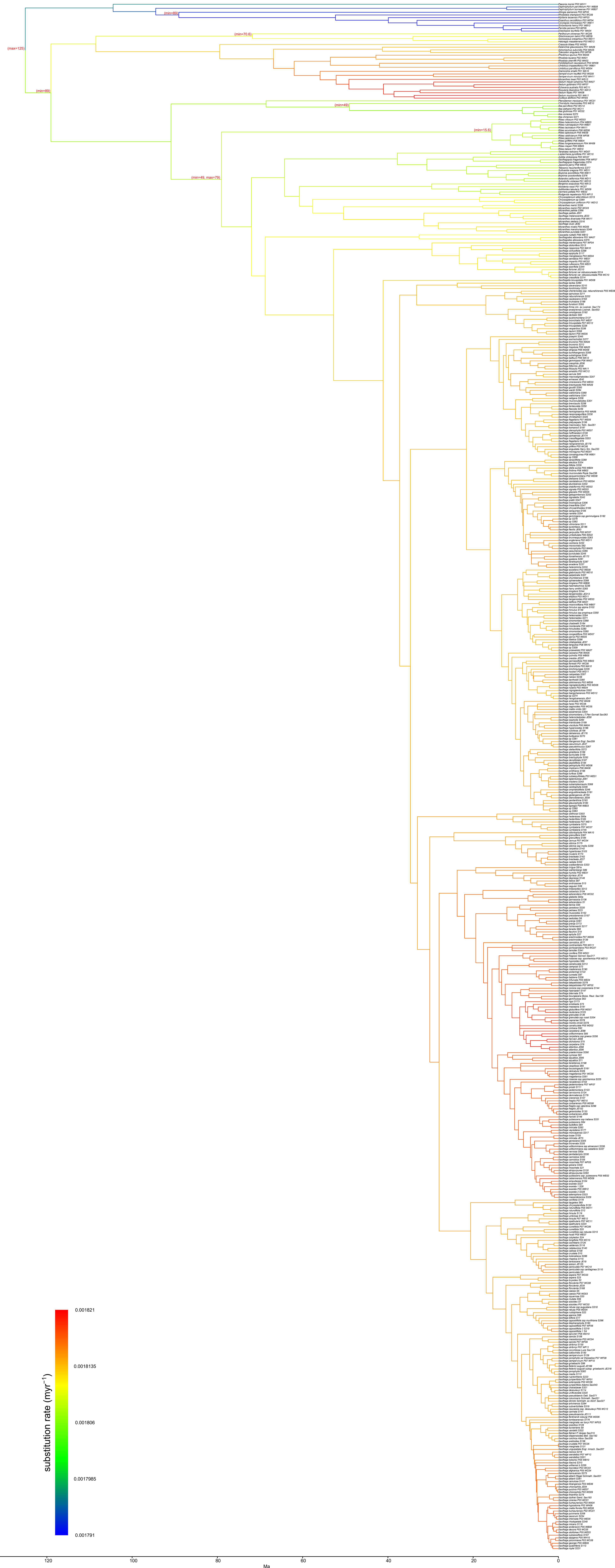

Supplementary Figure 2d

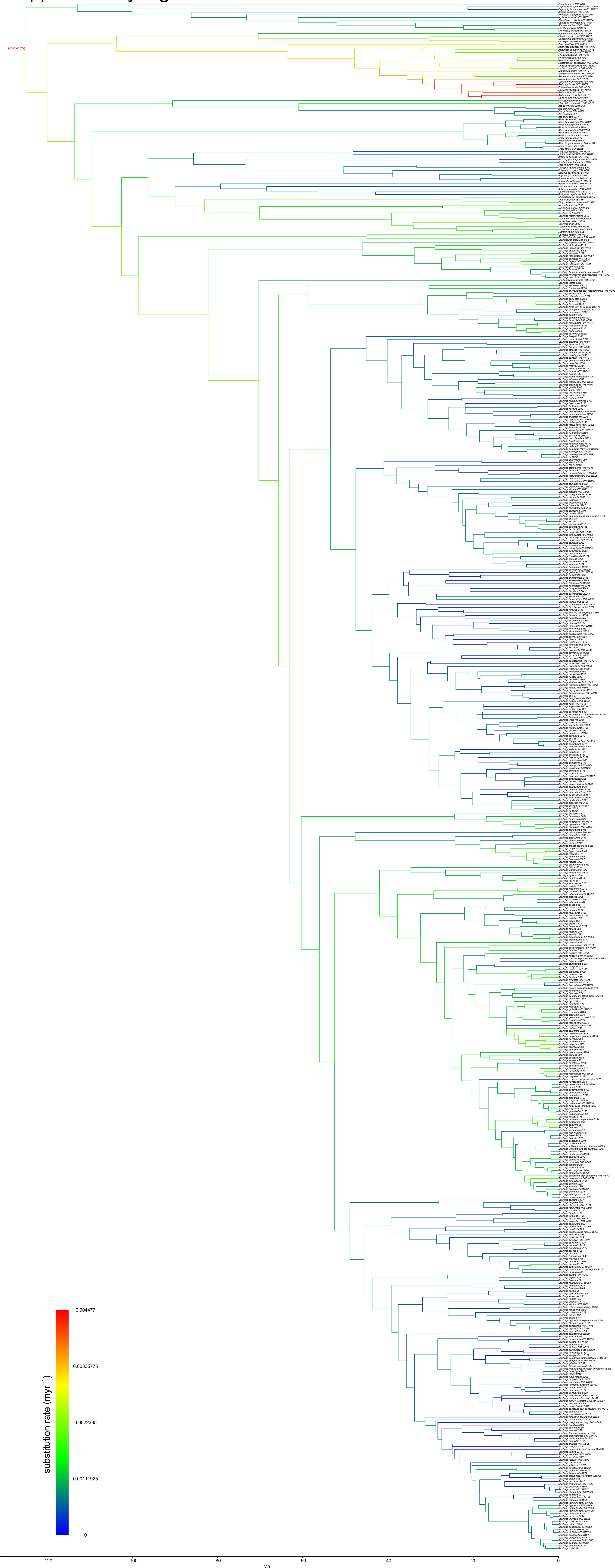

Supplementary Figure 2e

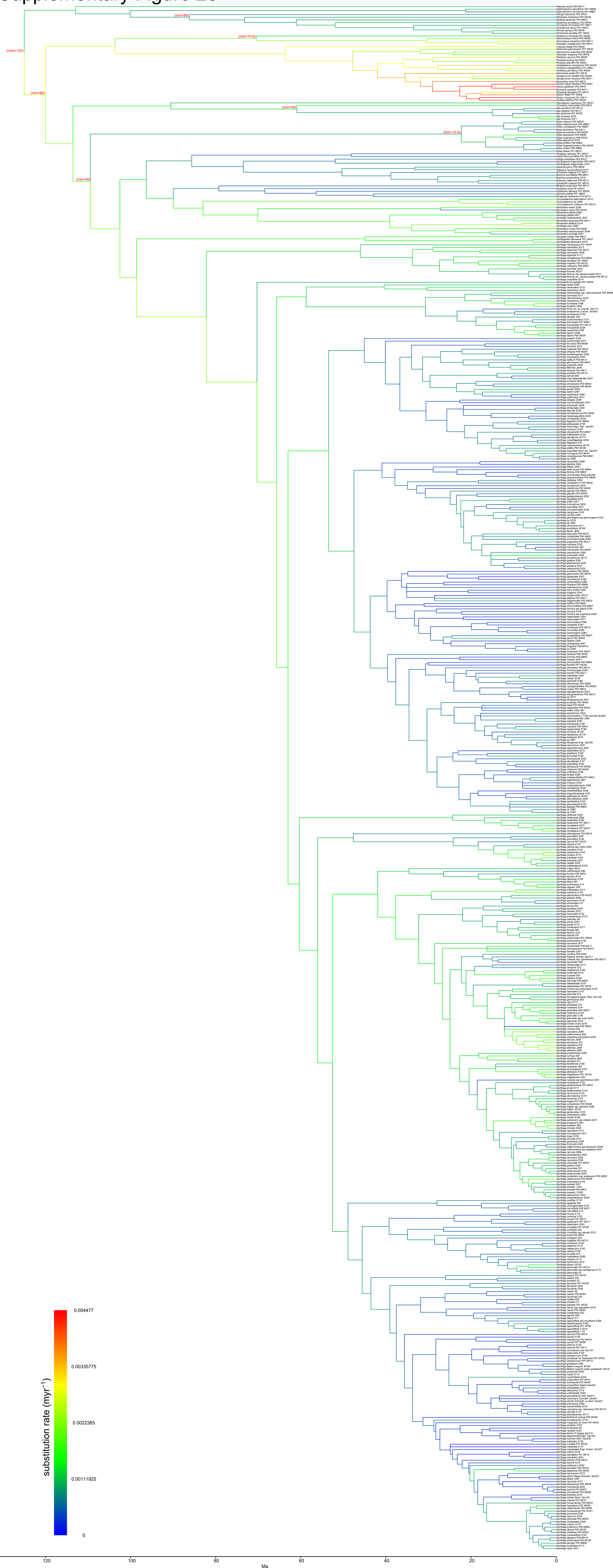

Supplementary Figure 2f

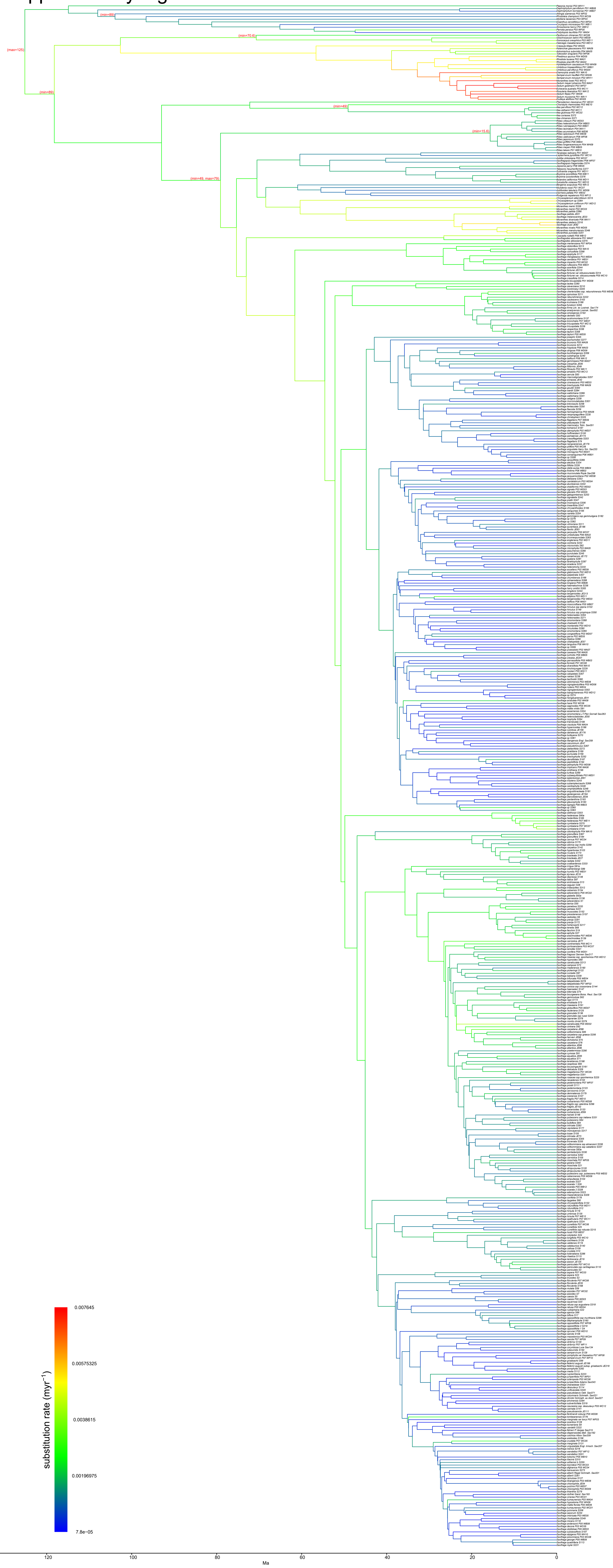

Supplementary Figure 2. Time-calibrated phylogenies for *Saxifraga* that were estimated in this study. Minimum and maximum constraints are shown where relevant. Branch colours refer to the estimated substitution rate for that branch. a) main, b) smoothing value of 0.01, c) smoothing value of 100, d) no internal constraints, e) no maximum constraints, f) gene shopping.

Supplementary Figure 3

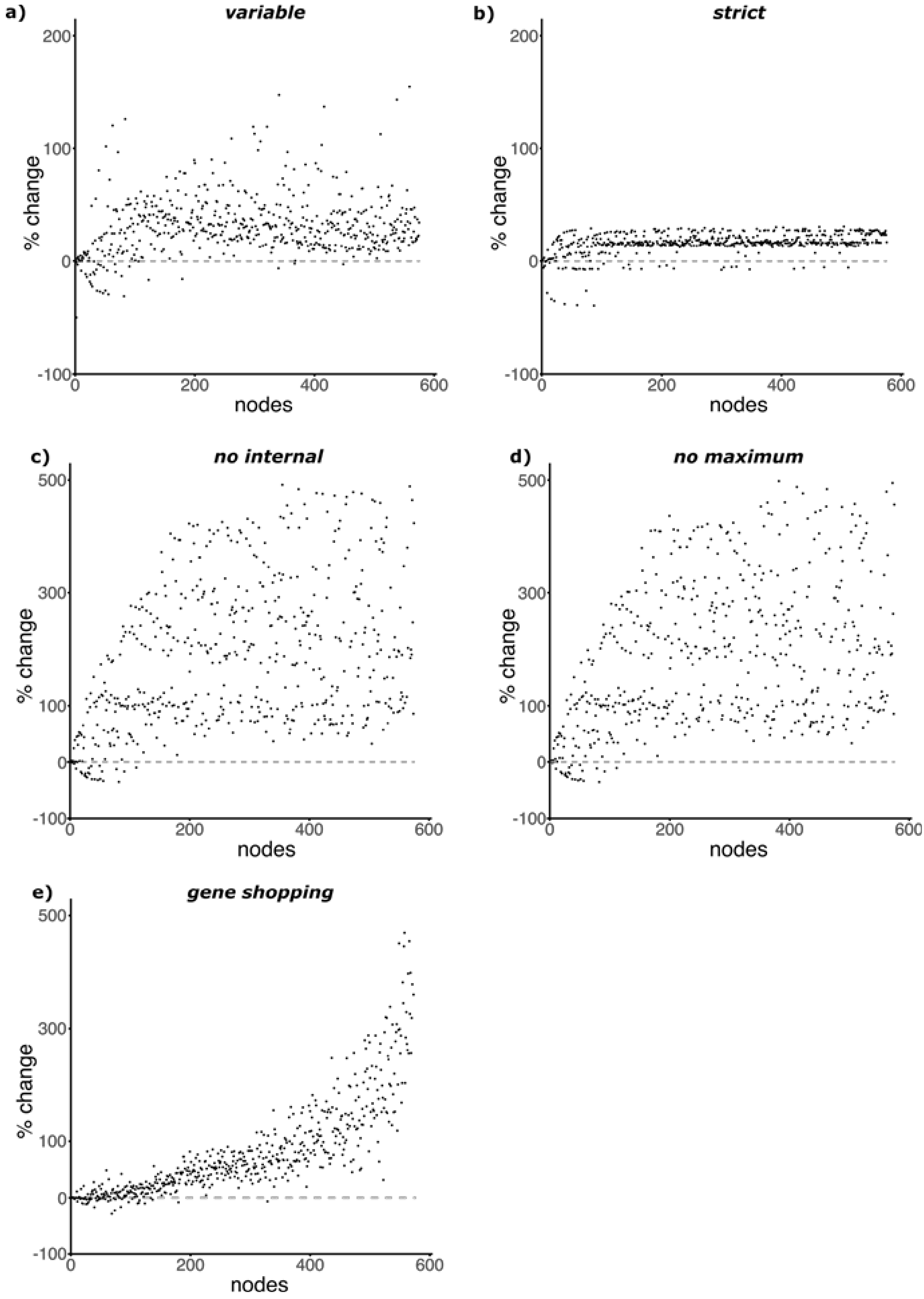

Supplementary Figure 3. Comparison of the age estimates for each node in the time-calibrated phylogenies, relative to *main*. Each plotted point refers to the percentage difference for each node in each time-calibrated phylogeny, relative to the age of the same node in *main*. a) smoothing value of 0.01 relative to *main*, b) smoothing value of 100 relative to *main*, c) no internal constraints relative to *main*, d) no maximum constraints relative to *main*, e) gene shopping relative to *main*.

Supplementary Figure 4

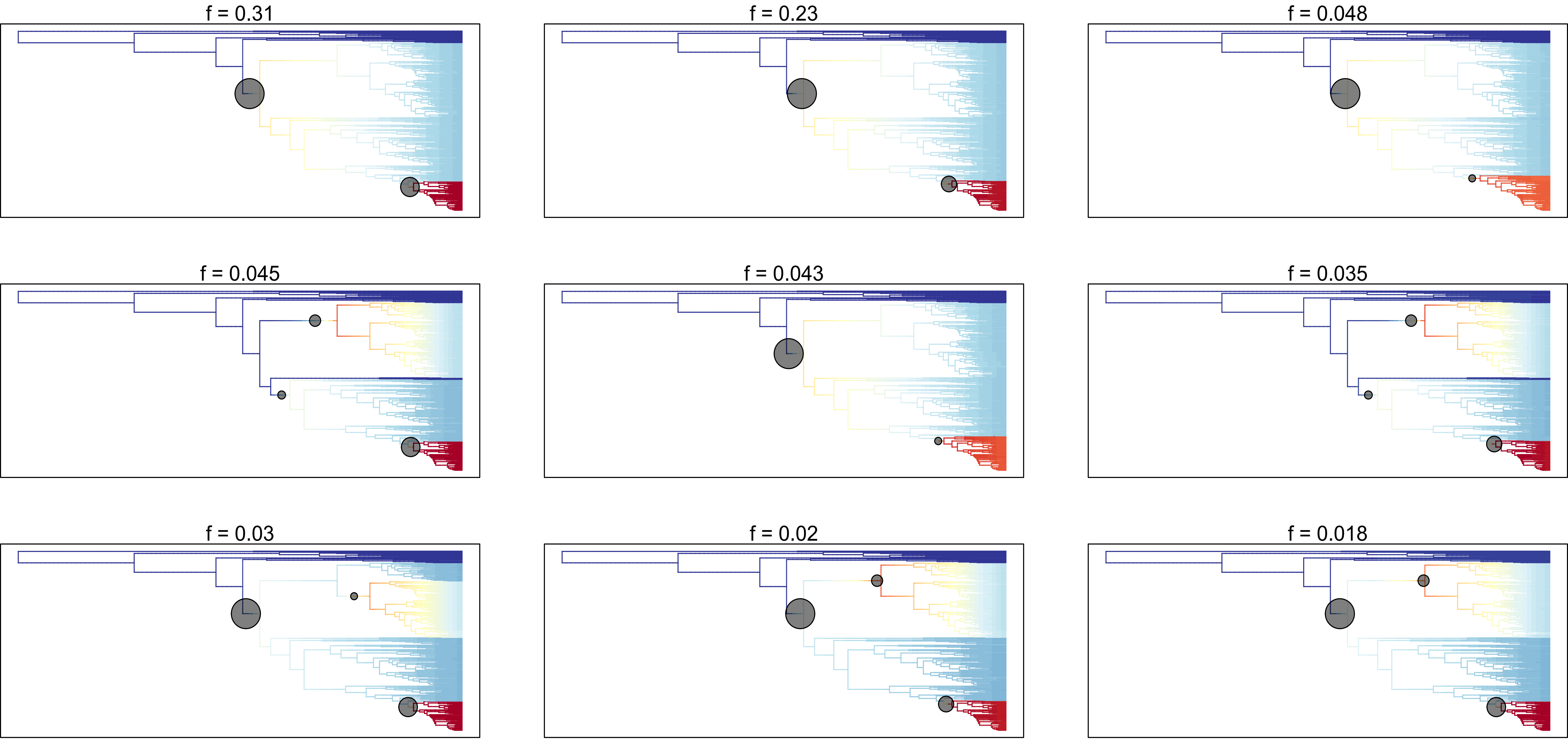

Supplementary Figure 4. Lineage-specific speciation rate estimation in BAMM on the time-calibrated phylogeny designated as *main*. The figure summarises the credible shift set which represents all the configurations of diversification rate shifts that incorporate 95% of the posterior probability.

Supplementary Figure 5

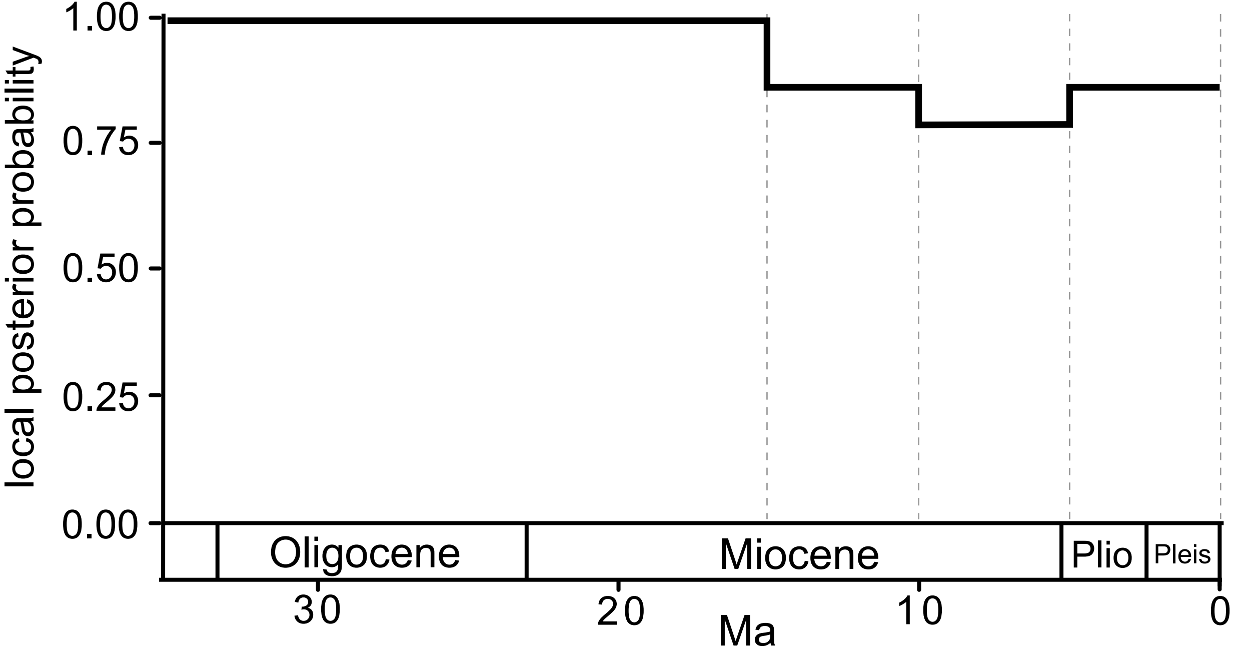

Supplementary Figure 5. Local posterior probability through time in the time-calibrated phylogeny designated as *main*. The plotted value is the mean local posterior probability for all nodes within each time interval. The same time intervals are used as in Figure 3a.

Supplementary Figure 6

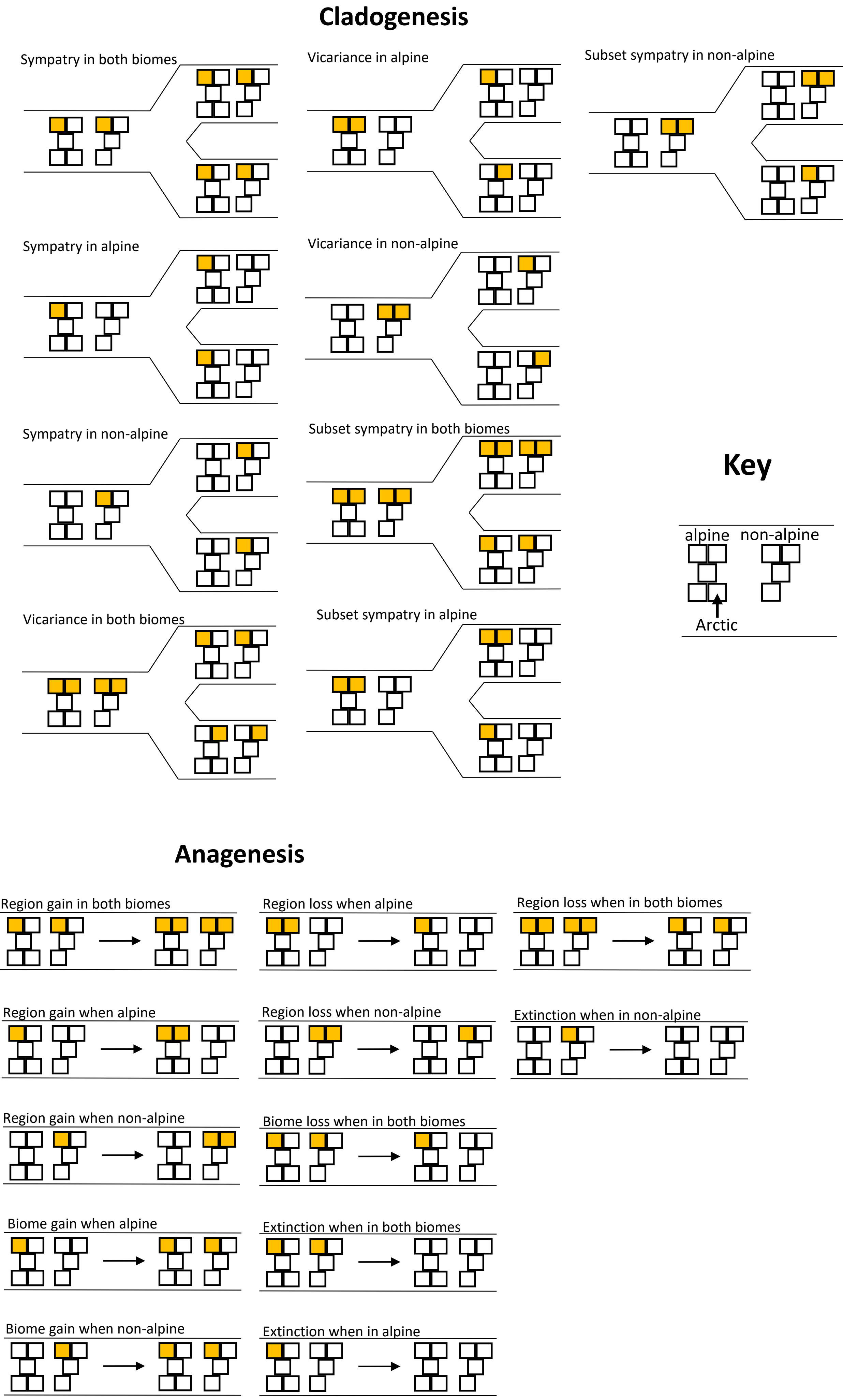

Supplementary Figure 6. Summary of the ClaSSE model used to perform biogeographic analyses. The 5 regions are indicated by the squares. Each region contains the alpine or non-alpine biome except for the arctic. Therefore, there are only four non-alpine regions, as indicated. A yellow filled square indicates the presence of a lineage in a region and biome. The *Anagenesis* and *Cladogenesis* diagrams summarise the processes that are incorporated into the model.

Supplementary Figure 7

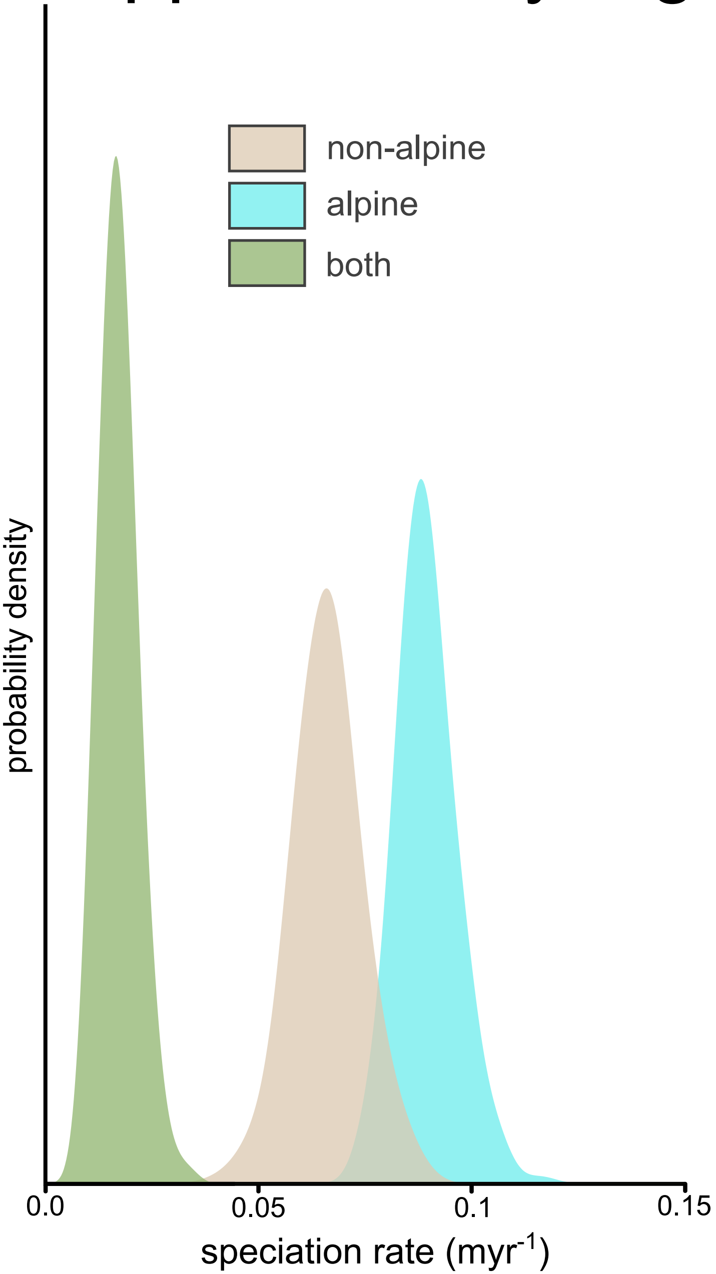

Supplementary Figure 7. Biome-specific speciation rate estimates from the ClaSSE model, using the time-calibrated phylogeny designated as *no maximum*.

Supplementary Figure 8

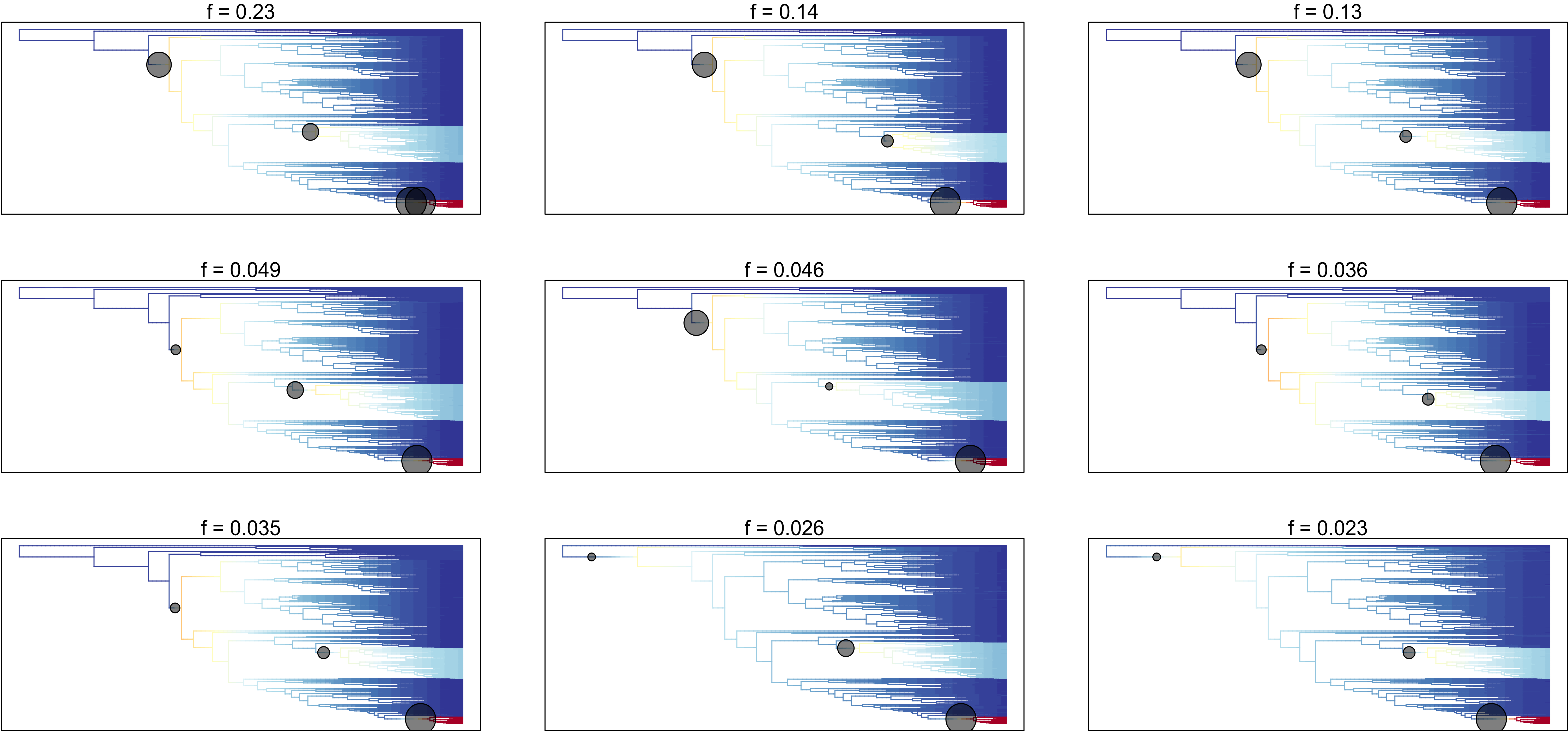

Supplementary Figure 8. Lineage-specific speciation rate estimation in BAMM on the time-calibrated phylogeny designated as *no maximum*. The figure summarises the *credible shift set* which represents all the configurations of diversification rate shifts that make up 95% of the posterior probability.

Supplementary Figure 9

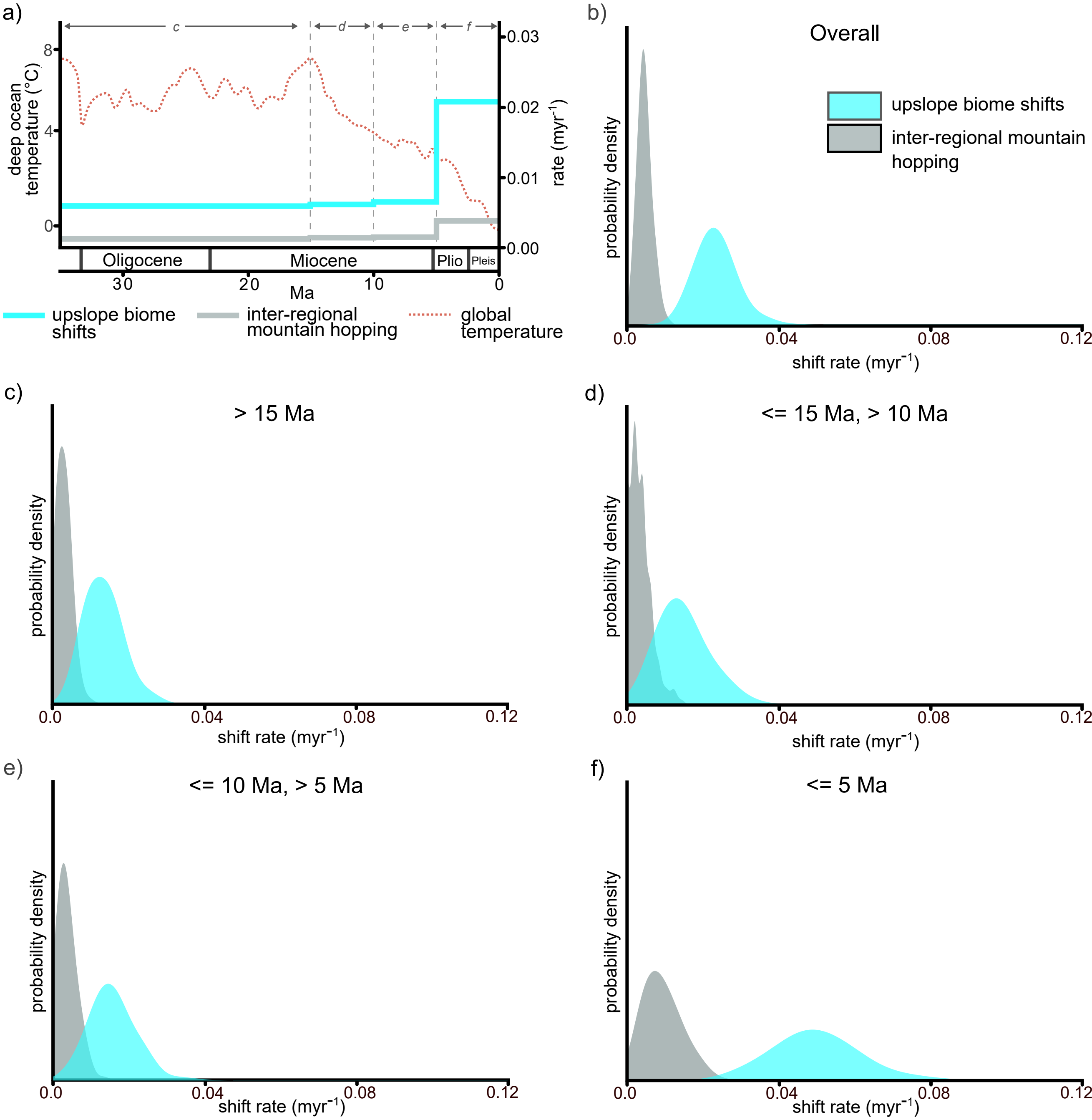

Supplementary Figure 9. Rates of upslope biome shifts and inter-regional mountain hopping in the time-calibrated phylogeny designated as no maximum. a) Posterior mean rate of upslope biome shifts and inter-regional mountain hopping through time, as well as global climate through time (estimated by deep-sea oxygen isotope records) [22]. The vertical dotted lines delimit the time intervals that are referenced in c-f. Plio = Pliocene, Q = Quaternary. b-f) Posterior distributions of rates of upslope biome shifts and inter-regional mountain hopping from the ClaSSE model, with b) showing rates across the entire time-calibrated phylogeny, c) showing rates at times over 15 Ma, d) showing rates 15 Ma or less, but over 10 Ma, e) showing rates 10 Ma or less, but over 5 Ma, and f) showing rates 5 Ma or less.

Supplementary Table 1. Summary of the different time-calibrated phylogenies that were estimated.

Name	Conflict		Time		Rate	
	congruent branches	congruent loci	minimum constraints	maximum constraints	autocorrelated	
					Cross-validation	Specified smoothing value
main	**Y**	**N**	**Y**	**Y**	**Y**	**N**
alternative smoothing value	Y	N	Y	Y	N	Y
no maximum	Y	N	Y	N	Y	N
no internal	Y	N	N	N	Y	N
gene shopping	N	Y	Y	Y	Y	N

* Y means a specific method was used, N means it was not used.

Supplementary Table 2. Summary of the different fossil calibrations used in this study.

Fossil	Age	Node	Reference
Flowers, fruits	89 Myr (Upper Cretaceous)	Iteaceae stem node	[1]
Flowers, fruit and pollen	89 Myr (Upper Cretaceous)	Altingiaceae stem node	[2]
Flowers, fruit	70.6 Myr (Upper Cretaceous)	Haloragaceae stem node	[3]
Pollen	49 Myr (early Eocene)	*Itea* stem node	[4]
Okinaga mountain specimen	49 Myr (early Eocene)	*Ribes* stem node	[5]
Ribes barrowsae	15.6 Myr	*Ribes* crown node	[5]

Supplementary Note 1

Results are discussed extensively in the main manuscript, and in Figures 1-3, and Supplementary Figures 1-5. Alongside the results already discussed, we also point out that results from analyses performed on the time-calibrated phylogeny designated as *no maximum* were broadly similar to results obtained with the time-calibrated phylogeny designated as main (Supplementary Figures 7-9). The only notable difference was that rates of alpine speciation were relatively higher in *no maximum* compared to *main*. Regardless, we consider the results obtained with *main* to be more reliable than those obtained with *no maximum*, for reasons outlined in the methods.

Supplementary References*

1. E.J. Hermsen, M.A. Gandolfo, K.C. Nixon, W.L. Crepet, *Divisestylus* gen. nov. (aff. Iteaceae), a fossil saxifrage from the Late Cretaceous of New Jersey, USA. *Am. J. Bot.* 90, 1373-1388 (2003).

2. Z. Zhou, W.L. Crepet, K.C. Nixon, The earliest fossil evidence of the Hamamelidaceae: Late Cretaceous (Turonian) inflorescences and fruits of Altingioideae. *Am. J. Bot.* 88, 753-766 (2001).

3. G.R. Hernández-Castillo and S.R. Cevallos-Ferriz, Reproductive and vegetative organs with affinities to Haloragaceae from the Upper Cretaceous Huepac Chert Locality of Sonora, Mexico. *Am. J. Bot.* 86, 1717-1734 (1999).

4. E.J. Hermsen, A review of the fossil record of the genus *Itea* (Iteaceae, Saxifragales) with comments on its historical biogeography. *Bot. Rev.* 79, 1-47 (2013).

5. E.J. Hermsen, The fossil record of Iteaceae and Grossulariaceae in the Cretaceous and Tertiary of the United States and Canada. *Cornell University Dissertations Publishing* (2005).

6. eFloras, Published on the Internet http://efloras.org [accessed 5 March 2022]. Missouri Botanical Garden, St. Louis, MO & Harvard University Herbaria, Cambridge, MA.

7. K. Magota et al., Phylogeographic analysis of *Saxifraga fortunei* complex (Saxifragaceae) reveals multiple origins of morphological and ecological variations in the Japanese Archipelago. *Mol. Phylogenetics Evol.* 163, 107230 (2021).

8. M. McGregor, Saxifrages: A definitive guide to the 2000 species, hybrids and cultivars (Timber Press, 2008).

9. K. Lauber and G. Wagner, Flora Helvetica (Haupt, 1996).

10. M. Saule, Nouvelle flore illustrée des Pyrénées (Éditions du Pin à crochets, 2018).

11. D.A. Webb and R.J. Gornall, Saxifrages of Europe (Christopher Helm, 2013).

12. M. Mannocci et al., Two new *Saxifraga* species (Saxifragaceae) endemic to Tuscan Archipelago (central northern Mediterranean, Italy). *Phytotaxa.* 248, 108-130 (2016).

13. P. Singh, K. Karthigeyan, P. Lakshminarasimhan, S.S. Dash. Endemic Vascular Plants of India (Botanical Survay of India, 2015).

14. G. Pils, Illustrated Flora of Albania (Christian Theiss GmbH, 2016).

15. G. Blanca, B. Cabezudo, M. Cueto, C. Salazar, C Morales Torres, Flora Vascular de Andalucía Oriental. (Universidades de Almería, Granada, Jaén y Málaga, Granada, 2011).

16. M. McGregor and W. Harding, Saxifrages. *The complete list of species* (The Saxifrage Society, Hutton, UK, 1998).

17. N. Tkach et al., Molecular phylogenetics, morphology and a revised classification of the complex genus *Saxifraga* (Saxifragaceae). *Taxon.* 64,1159-1187 (2015).

18. H. Sauerbier and W. Langer, *Endemische Alpenpflanzen: mehr als 500 Endemiten, 800 Fotos* (Margraf Publishers, 2017).

19. S. Pfanzelt, C. García, A. Marticorena, Notes on the Chilean geographic distribution of several vascular plant species. *Check List.* 9, 832-832 (2013).

20. T. Lafranchis and G. Sfikas, Flowers of Greece Vol. 1 (Diatheo, 2009).

21. Flora of Nepal online accounts, Published on the Internet http://www.floraofnepal.org/onlinef

*this list also includes references for Supplementary Data File 3.
