# Supplementary material for: Repeated upslope biome shifts in Saxifraga during late-Cenozoic climate cooling
Source: Nat Commun. 2024 Feb 6;15:1100. doi: 10.1038/s41467-024-45289-w (PMC10847498; doi:10.1038/s41467-024-45289-w)
Supplement: Supplementary file 3 — Description of Additional Supplementary Files [file 41467_2024_45289_MOESM3_ESM.pdf]

Description of Additional Supplementary Files

File Name: Supplementary Data 1
Description: Voucher information for samples in the study

File Name: Supplementary Data 2
Description: Information on samples included from Folk et al.

File Name: Supplementary Data 3
Description: Biome and regional preference for species.
